# The Future of Cross-Continental Telemedicine in the Management of Complicated Endocrine Patients and Its Suitability Based on a Case Report

**DOI:** 10.7759/cureus.22174

**Published:** 2022-02-13

**Authors:** Zahid Khan, Gideon Mlawa, Yousif Yousif, Abdullah Afghan, Dauda Balami, Mohammed Mohammed, SyedAun Muhammad, Vinod Warrier, Animesh Gupta, Maab Ibrahim

**Affiliations:** 1 Cardiology, Royal Free Hospital, London, GBR; 2 Internal Medicine and Diabetes and Endocrinology, Barking, Havering and Redbridge University Hospitals NHS Trust, London, GBR; 3 Internal Medicine, Barking, Havering and Redbridge University Hospitals NHS Trust, London, GBR; 4 Internal Medicine, Daisy Hill Acute Hospital, Newry, GBR; 5 Internal Medicine, Foxglove Multispecialty Hospital, Abuja, NGA; 6 Cardiology, Mid and South Essex NHS Foundation Trust, Southend on Sea, GBR; 7 Internal Medicine, Mid and South Essex NHS Foundation Trust, Southend on Sea, GBR; 8 Acute Internal Medicine, Barking, Havering and Redbridge University Hospital NHS Trust, London, GBR; 9 Internal Medicine, Sandwell and West Birmingham NHS Trust, Birmingham, GBR

**Keywords:** international collaboration and development, telehealth education, video telemedicine, telemedicine (tm), transphenoidal surgery, pituitary adenoma. brain tumor, emergency neurosurgery

## Abstract

Telemedicine is rapidly evolving to provide increased access to high-quality healthcare, and it has gained more traction during the current COVID-19 pandemic. Telemedicine was mostly restricted to remote areas, but with the COVID-19 pandemic, it has been adopted by hospitals and its use has increased significantly. In addition, international collaboration has also increased, and we present a case report from Nigeria whereby a patient was diagnosed with a pituitary tumor through telemedicine, and he underwent successful surgery. This case report highlights the opportunity for collaboration beyond borders and for health care professionals to work with developing countries to improve patients’ care.

## Introduction

Telemedicine describes the provision of medical services remotely and therefore encompasses the use of telecommunications such as telephone calls and video links. The current healthcare landscape is undergoing significant changes, particularly during the recent COVID-19 pandemic, and telemedicine has achieved a prominent role in health care. This is further supported by the recent technological advances and external pressures that have made telemedicine an attractive approach to modern medicine. The Centers for Medicare and Medicaid Services (CMS) made a declaration in 2020 by announcing the need for providers to provide patients' care in hospitals, clinics, and elsewhere by using telemedicine [1]. This has gained even more traction with newer and better safety policies to protect patients’ confidentiality [1]. Telemedicine is also cost-effective and has been proven to cost less for health care systems compared to face-to-face appointments for patients [2].

Telemedicine is revolutionary and offers additional benefits and easily accessible health care to patients with serious physical disabilities, elderly and frail patients, and patients in rural areas without access to the local hospital [3]. It has widened communication between doctors and patients from local to national and international levels, thus eliminating the relevance of distance. Previous studies have found telemedicine to improve accessibility to several specialties in a cost-effective manner by eliminating the need for travel [4,5]. For example, attendance at GP clinics has improved significantly after the introduction of app-based telemedicine services [6]. In the global health setting, telemedicine has been pivotal in providing new avenues for the remote provision of specialist expertise in low-resource settings, where specialist clinicians may be scarce [4,5]. The COVID-19 Pandemic has shed light on the fact that international collaboration, coordination, and collective responsibility are vital elements to the successful management of both communicable and non-communicable diseases [4].

## Case presentation

A 63-year-old Nigerian man presented with a year-long history of general malaise, weakness, weight gain, impaired sexual function, and impaired vision in the left eye. His past medical history included hypothyroidism post-total thyroidectomy, rheumatoid arthritis, hyperlipidemia, and hypertension.

**Figure 1:**
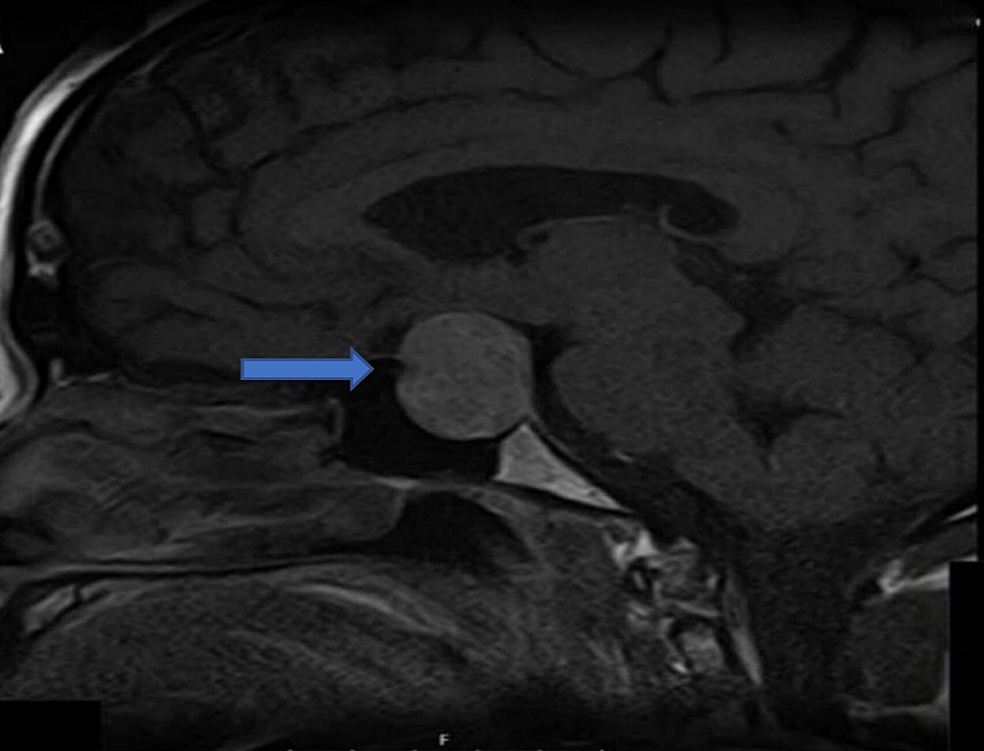
MRI head showing pituitary tumor

**Figure 2:**
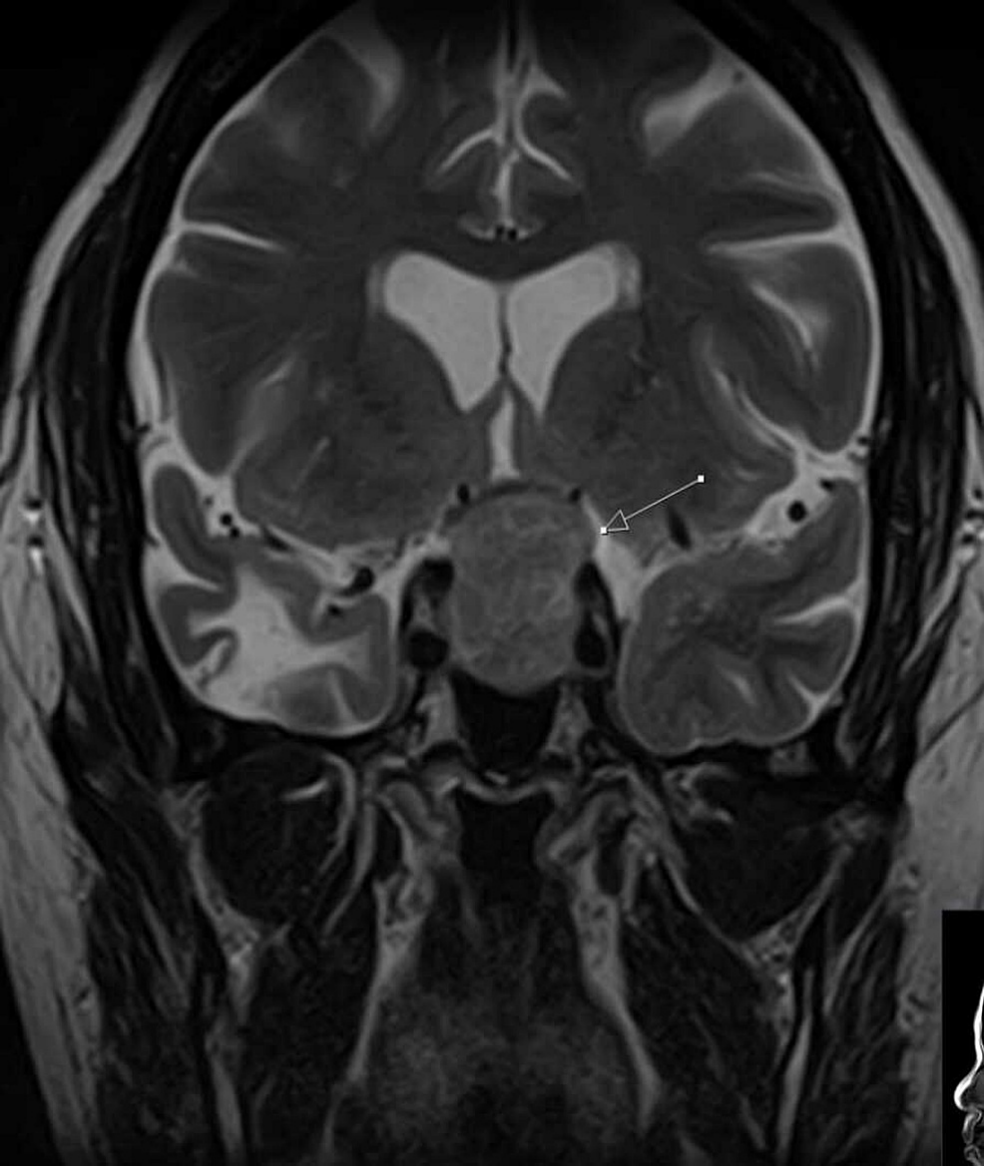
MRI shows pituitary tumor

**Figure 3:**
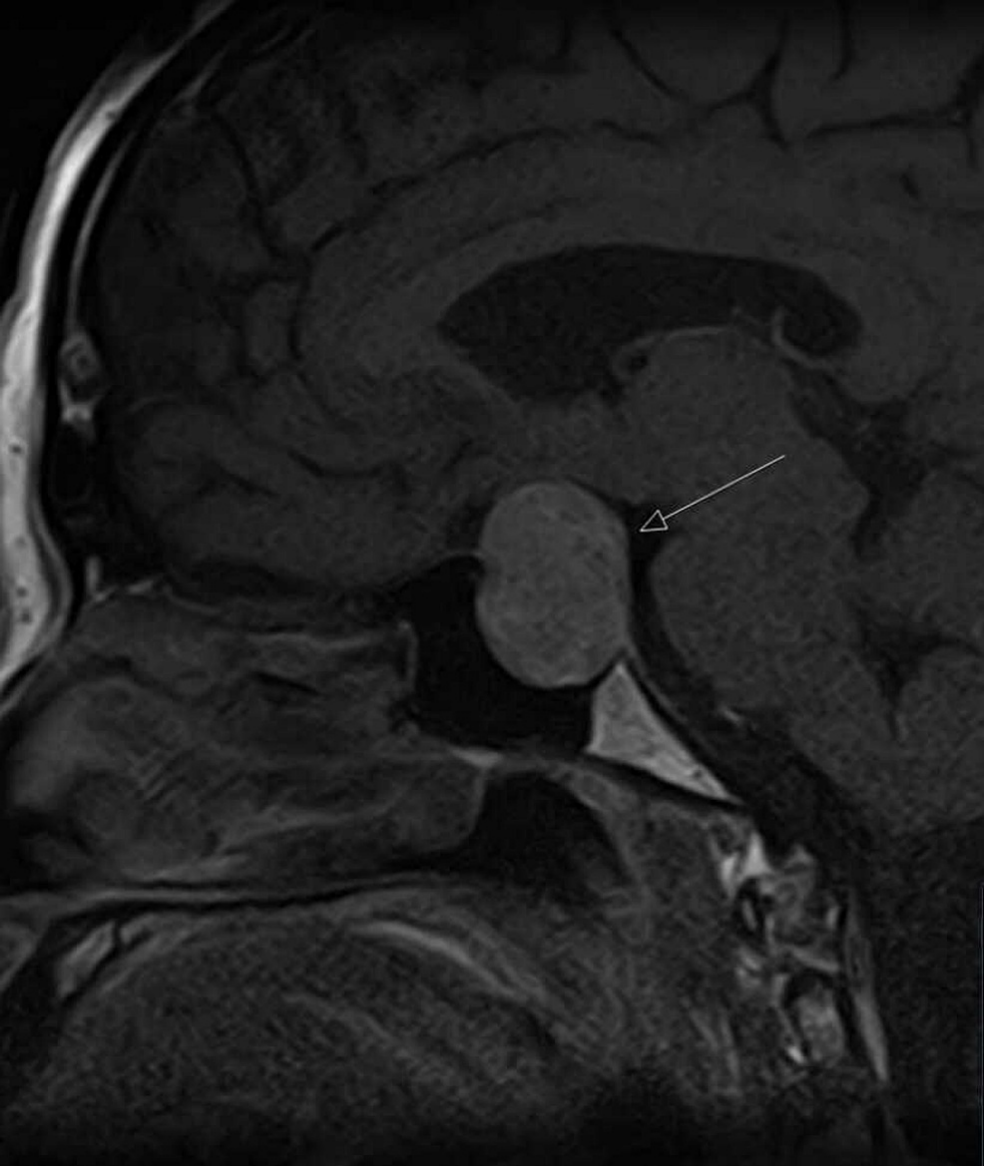
MRI showing pituitary tumor

Uncertain regarding the diagnosis, the Nigerian-based clinician responsible for patient care consulted with the physicians based at Queen’s Hospital, Romford, United Kingdom, using telecommunication with anonymized data. We were able to consult with the patient directly with patient consent and advised him to conduct a hormonal pituitary profile, initiate hydrocortisone therapy, and do magnetic resonance imaging (MRI) of his brain. The MRI brain showed a large 2.7cm diameter suprasellar pituitary adenoma (Figures 1-3).

**Figure 4:**
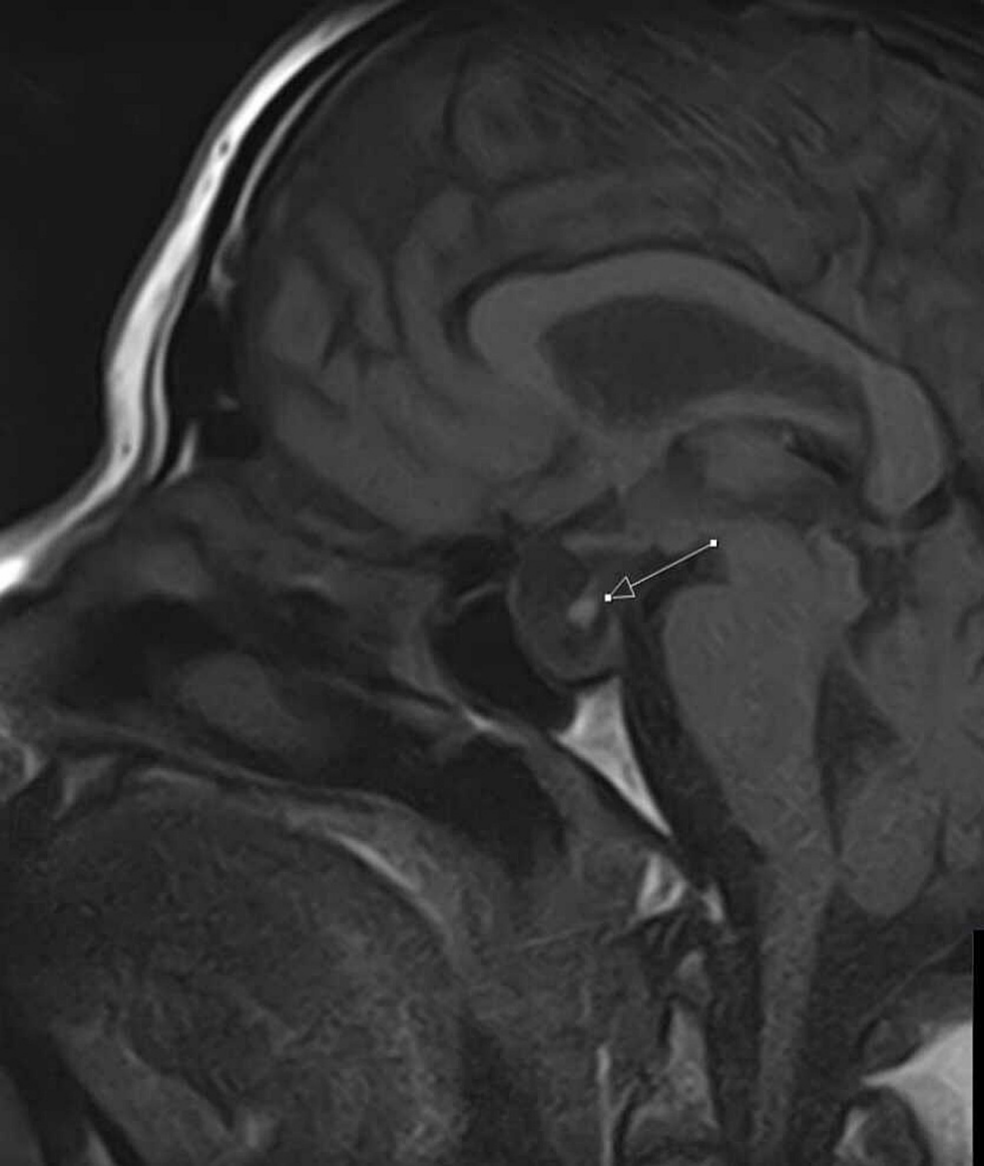
MRI head post transsphenoidal surgery shows a very small residual tumor

**Figure 5:**
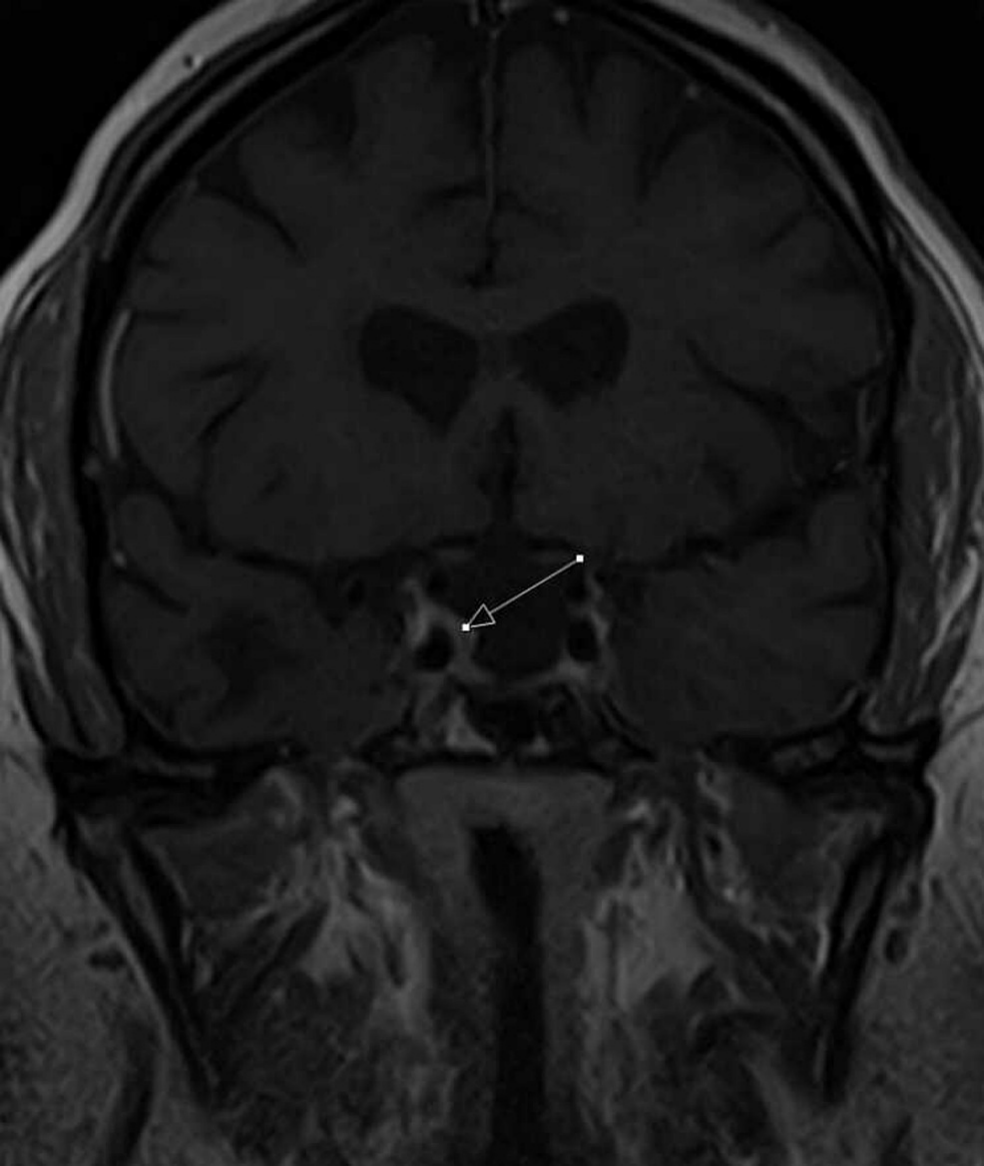
MRI shows small residual pituitary tumor

His lab results showed values consistent with secondary hypothyroidism, low morning cortisol of 100 nmol/L (normal value: 119-618 nmol/L), and low testosterone levels, and the patient was diagnosed with non-functioning pituitary adenoma. The thyroid-stimulating hormone level was 0.4 mU/L (normal value: 0.5-5.0 mU/L), and T4 was 4 μg/dl (normal value: 5-12 μg/dl). Given the visual disturbances, the patient was subsequently able to undergo transsphenoidal pituitary resection at Queen’s Hospital in October 2016, and now remains stable on hormonal replacement with hydrocortisone, levothyroxine, and testosterone (Figures 4, 5).

The patient has since returned to Nigeria and has been followed up annually by the team based at Queens Hospital, and he has remained stable on his current treatment.

## Discussion

**Figure 6:**
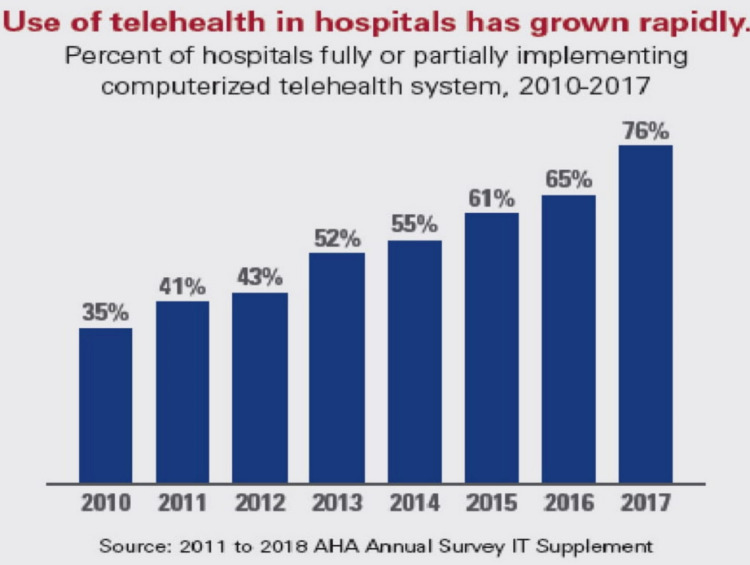
Telemedicine utilization in the US Hospitals Permission has been granted by American Hospital Association to use this figure [10].

Telemedicine has been used in rural communities, mainly in most countries where local community hospitals have been gradually reduced in number [6]. In addition, telemedicine has been incorporated into hospital outpatient clinics as well, and more and more patients are being offered telephonic appointments [7]. It is also worth mentioning that many patients these days are leaning towards virtual clinics if possible and are keen to avoid physical visits if possible [7]. In addition, telemedicine is rapidly evolving during the current COVID-19 pandemic and can provide access to high-quality and cost-effective care. Although some people use the terms "telemedicine" and "telehealth" interchangeably; however, this should not be the case as the two are different in various aspects. Telehealth can be considered as a broader concept of telemedicine that includes the use of telecommunication and information technology (IT) for remote monitoring of patients and delivering healthcare to them [8]. The utilization of telemedicine in United States (US) hospitals has increased from 35% to 76% from 2010 to 2017 (Figure 6), and the American Medical Association has reported that telemedicine insurance claims have increased by 53% in one year from 2016 to 2017 [9].

**Figure 7:**
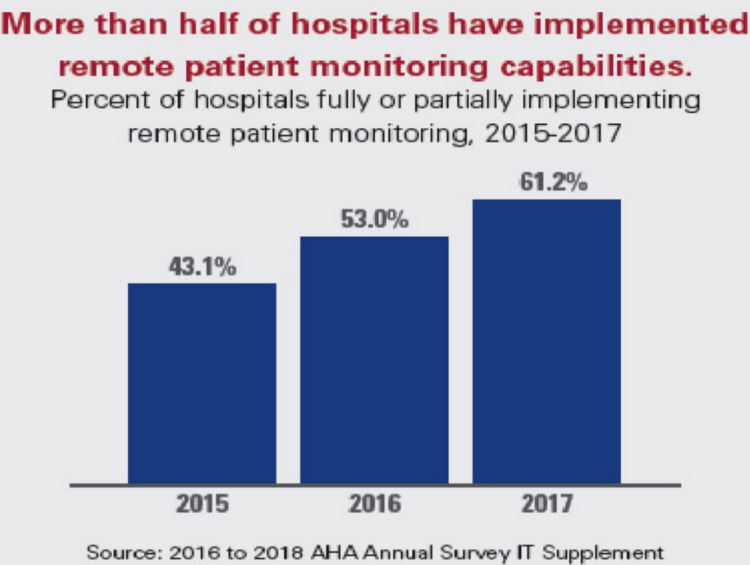
Remote patients monitoring facilities utilization in the US hospitals Permission has been granted by American Hospital Association to use this figure [10].

More than half of the US hospitals have implemented remote patient monitoring capabilities as shown in Figure 7 [9].

Telemedicine has been stratified and has expanded in various ways, and the greatest expansion has been in tele-stroke, and research has shown that certain medical specialties use it more than others [10]. When considering the use of telemedicine by different specialties, it has been documented that certain medical specialties use telemedicine more than others, and radiologists, psychiatrists, and cardiologists were reported to use telemedicine the most, at rates of 39.5%, 27.8%, and 24.1%, respectively, compared to immunologists, gastroenterologists, and obstetricians, who used it at rates of 6.1%, 7.9%, and 9.3%, respectively [10].

This case report demonstrates that international collaboration is a possibility between institutions based in low and high-resource settings and can facilitate the management of endocrine cases in a patient-centered way. This can improve outcomes and can be a model for management through global health collaboration, helping to meet growing healthcare demands in a time and cost-effective way that eliminates the need for travel for both patients and clinicians.

According to the Topol review, telemedicine is predicted to be the most disruptive technological advancement in healthcare between 2020 and 2040 [11]. This is partly because low-cost technology for collaboration is already available. However, there are regulatory issues that must be established surrounding information governance, privacy laws, and confidentiality [10,11]. One of the few silver linings of the COVID-19 pandemic is the rise of telemedicine. Telemedicine can increase access to healthcare for patients in remote rural areas with limited transportation and for immunocompromised patients to minimize their risk of exposure to infectious diseases [1].

Finally, previous studies have shown variable effectiveness of telemedicine-based interventions, dependent on the patient population and the nature of interventions provided [12]. However, several studies have shown no difference in therapy delivered over video-conferencing versus face-to-face care, although the former groups had higher dropout rates in earlier studies [13,14]. This may be reduced as access to portable devices, user interfaces, and training for telemedicine systems improves.

However, telemedicine is not without limitations, and the most significant limitation to its implementation is the lack of ability to check patients’ vital signs and perform physical examinations [1]. This can be partly overcome by providing patients with tools such as a weight scale, blood pressure cuff, pulse oximeter, and thermometer; however, it may still not be able to eliminate the inability to perform a physical examination.

A major concern among healthcare providers is the potential degradation of the patient-provider relationship, although a study at Dartmouth-Hitchcock Medical Center did not show any degradation in the relationship between physicians and patients involving telemedicine equipment [15,16]. When a telemedicine visit is not enough to address a patient’s concern, this can affect their satisfaction, and this limitation should be taken into consideration when interpreting data.

It is important to be aware of red flags in telemedicine and to make appropriate referrals when required. Few published case reports have highlighted this fact when red flags or warning signs have been ignored in patients and were advised superficial or symptomatic treatment only without arranging physical examination for them. One such case includes a patient who developed foot drop after developing acute onset back pain and was later found to have developed a large unilateral disc herniation with inferior migration at the appropriate level, warranting surgical consideration based on MRI lumbar spine [17,18].

A few other serious limitations of telemedicine include patients' data protection, insurance coverage, and the inability to provide emergency care. In addition, laws covering telemedicine vary across countries, and licensing can be an issue in certain countries. Finding the right technology to suit both patients and physicians can also be challenging.

## Conclusions

This case report demonstrates how patients in low-income countries may benefit from remote collaboration with specialists based in more economically developed countries through telemedicine, facilitating the democratization of healthcare. However, challenges lie in international differences in medical licensing, regulations around privacy and abuse laws, as well as the lack of establishment of appropriate technology in the most deprived countries.
